# Optimisation of *Mycobacterium bovis* BCG Fermentation and Storage Survival

**DOI:** 10.3390/pharmaceutics12090900

**Published:** 2020-09-22

**Authors:** Jordan Pascoe, Charlotte L. Hendon-Dunn, Colin P.D. Birch, Gareth A. Williams, Mark A. Chambers, Joanna Bacon

**Affiliations:** 1 TB Research Group, Public Health England, National Infection Service, Porton Down, Salisbury, Wiltshire SP4 0JG, UK; Jordan.Pascoe@phe.gov.uk (J.P.); c.hendon-dunn@nhs.net (C.L.H.-D.); 2 Department of Epidemiological Sciences, Animal and Plant Health Agency, Woodham Lane, New Haw, Addlestone, Surrey KT15 3NB, UK; Colin.Birch@apha.gov.uk; 3 Department of Bacteriology, Animal and Plant Health Agency, Woodham Lane, New Haw, Addlestone, Surrey KT15 3NB, UK; Gareth.A.Williams@apha.gov.uk (G.A.W.); m.chambers@surrey.ac.uk (M.A.C.); 4 Faculty of Health and Medical Sciences, University of Surrey, Guildford, Surrey GU2 7XH, UK

**Keywords:** BCG, fermentation, flow cytometry, calcein violet, sytox green, cryoprotectant

## Abstract

*Mycobacterium bovis* Bacillus Calmette–Guérin (*M. bovis* BCG) was generated over a century ago for protection against *Mycobacterium tuberculosis* (Mtb) and is one the oldest vaccines still in use. The BCG vaccine is currently produced using a pellicle growth method, which is a complex and lengthy process that has been challenging to standardise. Fermentation for BCG vaccine production would reduce the complexity associated with pellicle growth and increase batch to batch reproducibility. This more standardised growth lends itself to quantification of the total number of bacilli in the BCG vaccine by alternative approaches, such as flow cytometry, which can also provide information about the metabolic status of the bacterial population. The aim of the work reported here was to determine which batch fermentation conditions and storage conditions give the most favourable outcomes in terms of the yield and stability of live *M. bovis* BCG Danish bacilli. We compared different media and assessed growth over time in culture, using total viable counts, total bacterial counts, and turbidity throughout culture. We applied fluorescent viability dyes and flow cytometry to measure real-time within-culture viability. Culture samples were stored in different cryoprotectants at different temperatures to assess the effect of these combined conditions on bacterial titres. Roisin’s minimal medium and Middlebrook 7H9 medium gave comparable, high titres in fermenters. Flow cytometry proved to be a useful tool for enumeration of total bacterial counts and in the assessment of within-culture cell viability and cell death. Of the cryoprotectants evaluated, 5% (*v/v*) DMSO showed the most significant positive effect on survival and reduced the negative effects of low temperature storage on *M. bovis* BCG Danish viability. In conclusion, we have shown a reproducible, more standardised approach for the production, evaluation, and storage of high titre, viable, BCG vaccine.

## 1. Introduction

*Mycobacterium bovis* BCG (Bacillus Calmette–Guérin) was generated over a century ago for protection against *Mycobacterium tuberculosis* (Mtb) and is one the oldest vaccines still in use [1]. Though proven to be effective in reducing the mortality rate of Mtb, its variable efficacy has also been widely reported with some studies demonstrating that no significant protection was offered by the live vaccine [2,3]. The root cause of this variable efficacy isn’t entirely known with evidence suggesting that multiple factors can affect the potency of the vaccine, from the age of vaccination to variations in BCG vaccine strains used. In terms of production, variations in batches between, and even within, production centres has been highlighted as a key issue [3,4,5,6]. The World Health Organization (WHO) has noted a need for standardising the production and quality assessment of the BCG vaccine to not only mitigate the variable efficacy seen, but to also allow for easier evaluation in clinical trials of “new” & “old” BCG vaccines [7,8]. The detail of BCG vaccine manufacture is commercially sensitive. However, it’s known that BCG is currently produced using a pellicle growth method [9], whereby *M. bovis* BCG is cultured in stationary flasks and a pellicle forms at the liquid-air interface. The pellicle is then homogenised by ball-milling, and lyophilised. The complexity of multiple culturing and processing steps has been challenging to standardise [10]. In addition, production times are lengthy (~21 days) and labour-intensive, leaving production centres unable to effectively respond to changes in demand or shortages [11,12,13]. A global shortage of BCG lasting months, occurred in 2014–2015, when technical issues halted production at a single site [11,12]. The pellicle production method also causes quality control issues, where bacterial aggregation means that reliably assessing cell titres is challenging and will often produce highly variable results [8,13,14]. Production centres for the BCG vaccine are subject only to the regulation of their respective National Regulator Agencies (NRA) with many countries importing and using the vaccine through mutual recognition agreements of the NRA or by proxy through the United Nations Children’s Fund (UNICEF) [12]. Most NRA impose little regulation in how the BCG vaccine must be produced, apart from culturing for no more than 21 days by surface of submerged culture. End-point assessments, are required, such as the determination of the number culturable particles, vaccine efficacy, and, a lack of virulence in animals (usually guinea pigs) [7,15] [Personal Correspondence with MHRA, EMA, and DKMA]. Despite, very few imposed regulations, the validation of new approaches would be required prior to their licensure. Previously, it has been shown that more efficient culture methods, such as shaking cultures in batch flasks or batch fermentation, consistently produced a high titre of *M. bovis* BCG and provided comparable protection in animals, thereby posing a viable alternative to current BCG production methods [13,16]. Fermentation for BCG vaccine production would reduce the complexity associated with pellicle growth and should increase batch to batch reproducibility. Previous and current studies have shown that fermentation of *M. bovis* BCG is feasible and that BCG vaccine production in liquid culture affords an equivalent level of protection to that grown through pellicle-production [13,16].

We recently found that BCG vaccine produced by fermenter-growth gave protection to European badgers (*Meles meles*) against *M. bovis* infection [17]. This provided a foundation to further explore which fermentation conditions and storage conditions give the most favourable outcomes in terms of the yield of viable bacilli and stability of BCG. Here, we compare several different media, and assess growth over time in culture, using total viable counts, total bacterial counts, and turbidity throughout culture for twelve days in batch fermenters. We have also applied fluorescent viability dyes and flow cytometry to measure real-time within-culture viability. Samples taken throughout the culture were stored in different cryoprotectants at different temperatures to assess the effect of these combined conditions on bacterial titres.

## 2. Materials and Methods

### 2.1. Bacterial Strain

Master stocks of *Mycobacterium bovis* BCG (Bacillus of Calmette and Guérin) Danish strain 1331 (BCG) were prepared by spreading *M. bovis* BCG from an SSI vaccine stock vial (Staten Serum Institute, Copenhagen, Denmark) onto Middlebrook 7H10 supplemented with 10% oleic acid/albumin/dextrose/catalase (OADC) and incubating for three weeks at 37 °C.

### 2.2. Media

Six media were evaluated: Sauton’s minimal medium (Sauton’s), Roisin’s minimal medium (Roisin’s), Middlebrook 7H9 containing 10% OADC (Middlebrook), Dorset-Henly, CAMR *Mycobacterium* medium (CMM MOD2) [18], and Prosakeur-Beck. (All media were prepared by the authors at PHE-Porton). All media contained 0.5% glycerol and 0.2% Tween-80, which acted as a detergent. Antifoam emulsion C (Sigma, St Louis, MO, USA) was added to the media used in the fermenter batch culture to a final concentration of 0.05% (*v*/*v*). The full composition of each medium is described in Supplementary Tables S1–S8.

### 2.3. Reagents

The fluorescent dyes, calcein violet-AM (CV-AM) and sytox green (SG), were prepared in the following way. Each aliquot of calcein violet (catalogue no. C34858; Invitrogen, Life Technologies; Carlsbad, CA, USA) was dissolved in 25 µL dimethyl sulfoxide (DMSO) and then used immediately. SG (catalogue no. S7020; Invitrogen, Life Technologies, Carlsbad, CA, USA) was diluted from the manufacturer’s stock solution of 5 mM to a working solution of 20 µM in DMSO to be stored at −20 °C. Fresh aliquots were thawed and used on each occasion.

### 2.4. Flask Bacterial Culture

50 mL of each medium was placed in a 250 mL Erlenmeyer vented flask. The inoculum was prepared from *M. bovis* BCG grown on 7H10 agar containing 10% OADC agar grown for 3 weeks at 37 °C. Cultures were then incubated at 37 °C, and shaken at 200 RPM, for 14 days.

### 2.5. Culture of M. bovis BCG in Fermenters

The cultures were established following a modification of the method described previously [19]. Culture experiments were performed in 1-litre glass vessels at a working volume of 800 mL. The culture was agitated by a magnetic bar placed in the culture vessel coupled to a magnetic stirrer positioned beneath the vessel. Culture conditions were continuously monitored by an Anglicon Microlab Fermentation System, Eycoferm 4 (Brighton Systems, Newhaven, UK), linked to sensor probes inserted into the culture through sealed ports in the top plate. The vessel was filled with 800 mL of sterile culture medium (either Roisin’s or Middlebrook) and parameters allowed to stabilise at 37 °C, and pH 6.6. The vessel was placed on a magnetic stirring unit with a feed-back loop activated so that the magnetic stirrer in the culture would increase or decrease its speed (rpm) to control aeration of the culture to 50% dissolved air saturation (10% dissolved oxygen tension (DOT)). The oxygen concentration was monitored with a galvanic oxygen electrode (Broadly James, Silsoe, Bedford, UK). The inoculum was prepared from 7-day cultures of shaking flask-grown *M. bovis* BCG in the respective medium to be evaluated. The inoculum was standardised to an OD_600 nm_ of 1.0 in a volume of 40 mL. This 40 mL inoculum was introduced into the vessel via the sample port. Purity checks were performed on Typticase Soya Agar, No.2 Blood agar & Middlebrook 7H10 agar using a 100 µL of sample post-inoculation. The culture was controlled to 37 °C using a digital thermometer (Tempcon Instrumentation limited, Ford, Arundel, UK), and a heating pad positioned beneath the culture vessel. The culture was stirred at an agitation rate of approximately 500 to 750 rpm and the air saturation was maintained at 50% (10% dissolved oxygen tension). The initial culture acidity was set at pH 6.6 and was monitored throughout the experiment using an Ingold pH electrode (Mettler-Toledo, Leicester, UK). Each culture was maintained for 12 days and samples were removed regularly for further analyses.

### 2.6. Total Viable Counts and Culture Turbidity

To calculate total viable counts in shaking flask cultures, colony-forming units per mL (CFU mL^−1^) were determined using a modified Miles & Misra method; four dilutions were spotted in triplicate in 20 µL volumes on each agar plate [20]. Triplicate 100 µL samples were taken every 4 days during culture and a 10-fold serial dilution was performed in phosphate buffer solution (PBS) at pH 7.4 on each sample. Four dilutions were spotted in triplicate onto a single Middlebrook 7H10 agar containing 10% OADC [20]. The agar plates were incubated for three weeks at 37 °C and then colonies enumerated to calculate the total viable count (CFU mL^−1^). For the fermenter cultures, a 1 mL sample was taken every 24 h when possible for 12 days to measure turbidity at OD_600 nm_. To calculate total viable counts, 300 µL samples were also taken every 24 h and three individual 10-fold dilutions series were performed in PBS. These dilutions were then plated in triplicate onto 7H10 agar containing 10% OADC. These were incubated for three weeks at 37 °C and colonies were enumerated.

### 2.7. Viability and Titre of M. bovis BCG Using Flow Cytometry

The fluorescent labelling of *M. bovis* BCG was performed using the method previously described by Hendon-Dunn et al., 2016 [21]. Culture samples (100 µL) were placed in a 96-well plate (Corning, NY, USA) containing dyes, SG, (20 µM in 1 µL) & CV-AM (0.5 µL). Wells containing cells with a single dye, or no-dye, were also tested as controls. The samples were mixed with the respective dyes, and then incubated statically at 37 °C for 1 h, in the dark. The plate was spun at 2804× *g* for 5 min, the supernatant removed, and the pellet resuspended in Hank’s bank salt solution (HBSS) containing 4% formaldehyde (*v/v* in water) for fixation. The bacteria were fixed at room temperature in the dark for 1 h prior to analysis by a Beckman-Coulter S Cytoflex flow cytometer (Beckman Coulter, Brea, CA, USA). The bacterial population gate was set by forward-scatter and side-scatter to capture the single cell population. The Median Fluorescence (MF) was calculated for this population and for each dye. SG fluorescence emission was detected in the 530/40 BP channel, and CV-AM fluorescence was detected in the 450/50 BP channel. The bacterial titre was determined by the events/mL; this was calculated through the addition of a known quantity of fluorescent beads to unstained culture samples, thereby determining the ratio of events between the fluorescence beads and the single cell population using flow cytometry. This ratio allowed for the calculation of events/mL.

### 2.8. Impact of Storage Conditions on M. bovis BCG Viability

To assess the impact of different media, culture duration, storage temperature, and/or cryoprotectant on *M. bovis* BCG viability after 24 h of storage, 100 mL was harvested from the fermenter on day 4, day 7, and day 12 of culture. A 40 mL aliquot and two 30 mL aliquots were taken from each 100 mL sample. Each sample was spun at 3087× *g* for 5 min. Supernatant was removed from the 40 mL sample and the pellet was resuspended in 4 mL of a filter-sterilised 1.5% monosodium glutamate (MSG) (*w/v* in water). The two 30 mL samples were also spun at 3087× *g* for 5 min and supernatant removed. One of the sample pellets was resuspended in 3 mL of 1.5% (*w/v*) MSG containing 5% DMSO (*v/v*); the other was resuspended in 3 mL of filter-sterilised 1.5% (*w/v*) MSG containing 10% glycerol (*v/v*). Triplicate samples of 100 µL were taken from each of the different cryoprotectant solutions to assess pre-storage total viable counts via a 10-fold dilution series, and enumeration was performed by a modified Miles and Misra method [20]. A total of 300 µL was removed, and cryoprotectant solutions were stored in duplicate at +4 °C, −20 °C, and −80 °C. These aliquots were taken out of storage after 24 h and thawed at room temperature for 30 min. A 10-fold dilution series was performed for each aliquot and then plated in triplicate via the Miles & Misra method [20]. Agar plates were incubated at 37 °C and colonies were counted after three weeks.

### 2.9. Statistic Analyses

The statistically significant differences between growth (total viable counts and total cell counts) in the different culture media were identified by transforming the data by log_10_ to achieve a normal distribution, followed by a one-way ANOVA in R-Studio [22]. Following this, the contribution of the different parameters (sampling day, temperature, medium, and cryoprotectant) to bacterial survival, were assessed. In the first instance, the changes in the total viable counts, pre- and post-storage, for all parameters were log-transformed and visualised. To perform the statistical assessments, Box-Cox analyses were performed on total viable counts (CFU mL^−1^), across all parameters, to identify the optimal transformation to achieve a normal distribution. As a result of this assessment, a cube-root transformation was adopted [23]. The changes in the total viable counts (CFU mL^−1^) between pre- and post-storage were calculated for the combined parameters. A factorial ANOVA was performed on these changes to assess whether individual parameters or interactions between parameters exhibited a statistically significant impact on *M. bovis* BCG stability. A Post-hoc Tukey’s honestly significant difference (HSD) test was then performed on the outputs from the Factorial ANOVA analyses for those parameters that had demonstrated a significant impact on bacterial survival. Multiple pair-wise comparisons identified which conditions (e.g., temperature −20 °C, −80 °C, or +4 °C) were more beneficial for storage stability. All analyses described here were performed in R-Studio [22].

## 3. Results

### 3.1. M. bovis BCG Cultivation

#### 3.1.1. Roisin’s and Middlebrook Support Growth of BCG in Shaking Flasks

**Figure 1:**
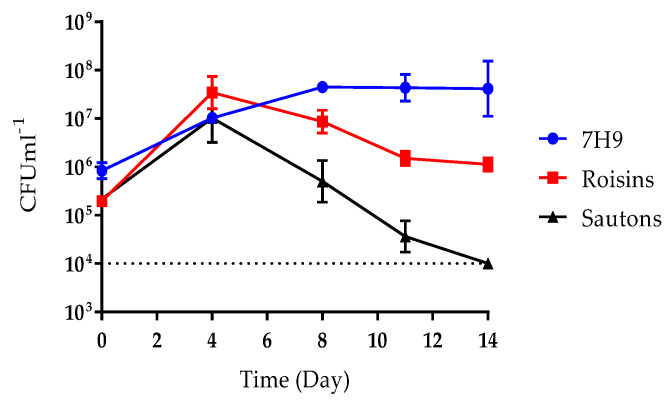
The total viable counts of *M. bovis* Bacillus Calmette–Guérin (BCG) flask grown in 50 mL of Middlebrook 7H9, Roisin’s minimal medium, or Sauton’s minimal medium, over 14 days. Culture in 250 mL vented Erlenmeyer flasks shaking at 200 RPM at 37 °C. 100 µL samples were taken, a 10-fold dilution series performed and spotted onto 7H10 + 10% OADC agar using the Miles and Misra method. Plates were incubated for three weeks at 37 °C and then colonies enumerated. The dotted line represents the limit of detection (1 × 10^4^ CFU mL^−1^). Data represent the mean average of three biological repeats ± standard error.

To select media for *M. bovis* BCG fermentation, growth was first assessed in six different media in shaking batch cultures at 37 °C at 200 rpm for 14 days. Dorset-Henly, CMM MOD2, and Prosakeur-Beck were unable to support growth (Supplementary Figure S1), whereas Sauton’s, Roisin’s, and Middlebrook, all supported growth (Figure 1). Sauton’s produced a total viable count of approximately 1 × 10^7^ CFU mL^−1^ by day 4. However, after 4 days of culture, the total viable count fell rapidly to level that was below the limit of detection (1 × 10^4^ CFU mL^−1^), by day 14. Roisin’s and Middlebrook supported the more favorable growth than Sauton’s, with both media producing a higher viable count of approximately 5 × 10^7^ CFU mL^−1^ by day 4 and by day 8, respectively. A gradual fall in the titre of viable bacilli was also observed in Roisin’s to 1 × 10^6^ CFU mL^−1^ by day 14, but this was less substantial than observed in Sauton’s. The total viable counts in Middlebrook remained steady at around 5 × 10^7^ CFU mL^−1^ for the remainder of culture duration (Figure 1). Middlebrook and Roisin’s were selected for fermentation, as they produced the highest bacterial titre and were the only two media that demonstrated continued viability over 14 days of culture.

#### 3.1.2. Middlebrook and Roisin’s Produce Equivalent *M. bovis* BCG Titres in Batch Fermentation

**Figure 2:**
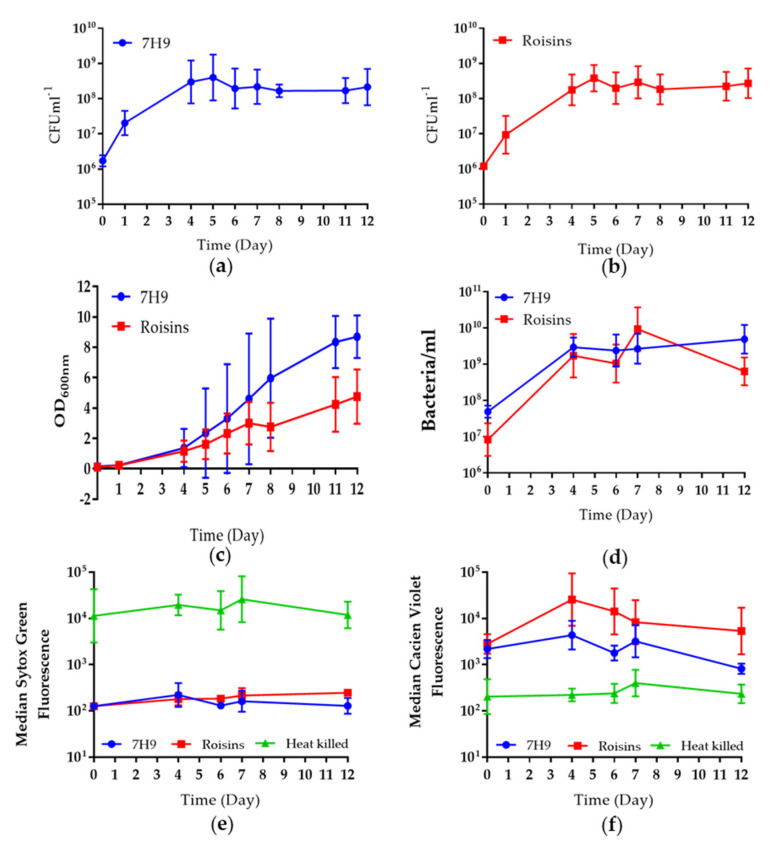
The total viable counts of fermenter-grown *M. bovis* BCG in 800 mL of either Middlebrook 7H9 (**a**) or Roisin’s minimal medium (**b**) over 12 days of culture in batch fermenters. The cultures were sampled daily for total viable counts (CFU mL^−1^) (**a**,**b**) and turbidity (**c**). Samples were taken on days 0, 4, 6, 7 & 12 for total bacterial counts (Bacteria/mL) determined using fluorogenic beads and flow cytometry (**d**). Samples were taken on the same days for analyses of cell death and metabolic state using flow cytometry and fluorescent dyes, sytox green (**e**) and calcein violet (**f**), respectively. A heat-killed *M. bovis* BCG control was included. Data represent the mean average of three independent culture repeats ± standard error.

The two media, Roisin’s and Middlebrook, were assessed for their ability to cultivate *M. bovis* BCG in batch fermenters, with the aim of identifying which medium gave sustained and optimal viability and the largest biomass, over 12 days of culture. Three independent replicate fermenter cultures were performed for each growth medium and samples were taken throughout, for total viable counts, and for measuring real-time viability using fluorescence dyes and flow cytometry. *M. bovis* BCG cultivated in either Roisin’s or Middlebrook demonstrated higher viable counts than their shaking flask counterparts (Figure 2a,b). *M. bovis* BCG reached peak viable counts by day 5 in both media and achieved titres of 4.0 × 10^8^ CFU mL^−1^ (±3.9 × 10^7^) and 3.8 × 10^8^ CFU mL^−1^ (±3.8 × 10^7^) in Middlebrook and Roisin’s, respectively. In both media, this high titre was maintained for the remainder of the culture with the average CFU mL^−1^ decreasing by less than 0.5 log_10_ CFU mL^−1^ by day 12. The growth of *M.bovis* BCG in the fermenters was similar in the two-growth media with no significant differences seen between the total viable counts gained (*p* value > 0.9999). Middlebrook supported less variable growth for the entirety of culture. However, in contrast to this, when peak titres were reached in culture, total viable counts were less variable in Roisin’s minimal than Middlebrook. Bacterial counts (Figure 2d) were marginally higher than the total viable counts, but there was no significant difference between these and the viable counts for the two culture media (*p* value 0.2654 and 0.5396 for Middlebrook and Roisin’s, respectively). This difference was likely due to the presence of cells that were dead or viable but not culturable on agar.

#### 3.1.3. Flow Cytometry Analyses of Culture Viability and Biomass, Show Comparable Growth in Roisin’s or Middlebrook

Flow cytometry was applied as an alternative approach to total viable counts to assess population viability and a bacterial count as predictors of total viable counts. The advantage of flow cytometry is that it can be performed on the same day as sampling, within the culture time-course, allowing for an assessment of culture viability to be made without waiting for three weeks post-culture to enumerate colony-forming units on agar. In addition to this, it provides useful information about the proportion of dead bacteria in the bacterial biomass, an important consideration for the efficacy of BCG vaccine. Total viable counts will only provide information about the number of bacteria that can grow on agar post-culture. Flow cytometry enabled us to gain an accurate measure of the total number of bacilli in culture and whether they were viable, viable, but non-culturable on agar, or dead. Fluorescence dyes, Sytox green (SG) and calcein violet (CV-AM) were used as previously described by Hendon-Dunn et al., 2016 [21] to measure cell viability and cell death, respectively. The CV-AM staining profile of fermenter-grown BCG indicated an increase in metabolic activity at the start of culture in each growth media, following which, the CV-profile remained steady until a reduction in median fluorescence by day 12; particularly in Middlebrook. The staining profiles indicated a lower metabolic activity throughout culture in Middlebrook. However, this difference was not statistically significant (Figure 2e,f; *p*-value ≥ 0.6622). Neither medium demonstrated a significant increase in SG-staining during culture, from their initial values of 189.9MFi (±90.8) in Middlebrook and 134.5MFi (±17.6) in Roisin’s. This indicates a pre-existing population of dead bacilli that did not increase significantly over the 12 days in culture.

#### 3.1.4. Optimal Conditions for Storage of *M. bovis* BCG were Cryopreservation in 1.5% MSG, Supplemented with 5% DMSO, and Storage at +4 °C

**Figure 3:**
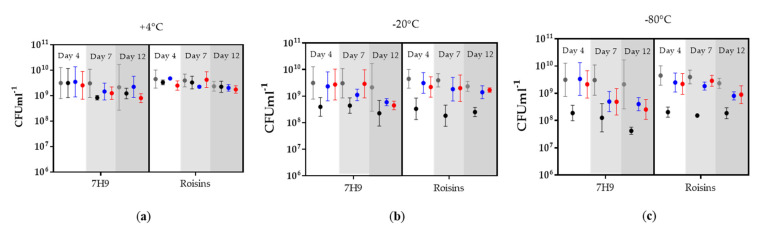
The total viable counts of *M. bovis* BCG cultured in Middlebrook or Roisin’s; pre-storage (•) and after 24h of storage at (**a**) +4 °C, (**b**) −20 °C, & (**c**) −80 °C in three different cryoprotectant solutions: (•) 1.5% MSG, (•) 1.5% MSG + 5% DMSO, (•) 1.5% MSG + 10% glycerol. Error bars represent the standard error on the mean average of three culture replicates.

The aims were to identify an optimal set of conditions for the storage of *M. bovis* BCG to mitigate the loss of viable bacilli that is often observed with storage of bacterial biomass, and to yield as high a *M. bovis* BCG titre as possible. We selected cryoprotectants that are commonly used in the preservation of bacterial culture, to evaluate them in combination with storage temperature, culture medium, and culture time-point, to determine whether quantitative differences to the survival of *M. bovis* BCG, treated under these different conditions, could be ascertained. Samples removed, after 4 days, 7 days, and 12 days, of growth, from cultures in two different media (Roisin’s or Middlebrook), were prepared in three different cryoprotectant solutions (1.5% MSG, 1.5% MSG supplemented with 10% glycerol or 1.5% MSG supplemented with 5% DMSO) and stored for 24 h at either +4 °C, −20 °C, or −80 °C. A timescale of 24 h was selected as previous data indicated that loss of viable bacteria occurred when stored at −20 °C and −80 °C within this time-period (Supplementary Figure S2). At +4 °C, loss of viable bacteria only began to occur when stored for longer than 3 months (Supplementary Figure S2). Total viable counts prior to storage were at approximately 10^9^–10^10^ CFU mL^−1^ (Figure 3). Even under the least beneficial storage conditions, the total viable counts remained above 10^7^ CFU mL^−1^. Despite both media producing comparable pre-storage total viable counts, Middlebrook demonstrated greater variability than Roisin’s (as shown by the error bars in Figure 2), which we surmise may have subsequently affected the variability of the post-storage bacterial titres.

**Figure 4:**
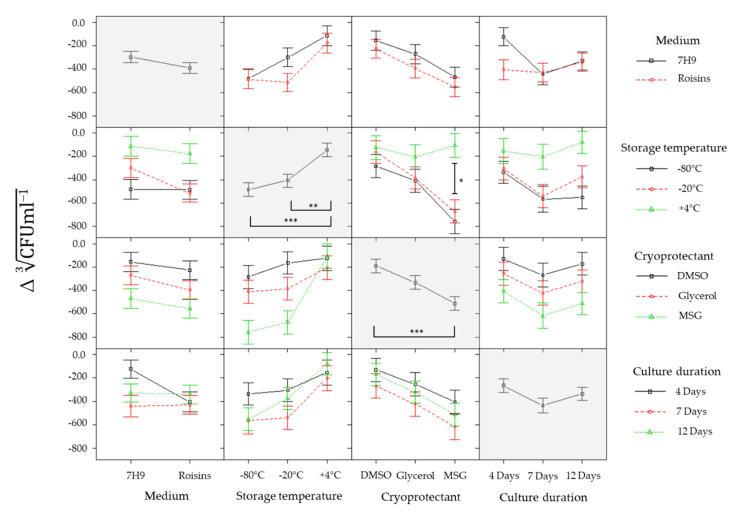
The change in *M. bovis* BCG total viable counts (cube root-transformation CFU mL^−1^), between fermenter culture samples that were exposed to different storage conditions and stored for 24 h. Shown here, is the effect of each condition on the change in *M. bovis* BCG total viable count and the interaction effects between combinations of two different conditions (white boxes). Starting titers prior to storage were 2.77 × 10^9^ (±4.41 log_10_ CFU mL^−1^) and 3.60 × 10^9^ (±2.64 log_10_ CFU mL^−1^) for culture in Middlebrook and Roisin’s, respectively. Error bars represent standard error on the mean average of three culture replicates. ‘***’ *p* = 0.001, ‘**’ *p* = 0.01 ‘*’ *p* = 0.05.

To assess the impact of temperature, culture medium, and/or cryoprotectant (defined here as “parameters”) on the loss of viable bacteria, the change in total viable counts for each individual biological replicate was calculated against its respective pre-storage value (Figure 3). From these data, the total viable counts were cube-root transformed and the changes in the total viable counts (CFU mL^−1^) between pre- and post-storage were calculated for the combined parameters (Figure 4). A Factorial ANOVA was performed on the changes in total viable counts (cube-root-transformed CFU mL^−1^) between pre- and post-incubation to assess whether individual parameters or interactions between parameters exhibited a statistically significant impact on *M. bovis* BCG stability under the conditions evaluated (Supplementary Table S9). A Post-hoc Tukey’s HSD test (99% confidence) was then performed for parameters that had significantly impacted bacterial survival in the factorial ANOVA to identify which conditions (e.g., −20 °C, −80 °C, or +4 °C), within a parameter (e.g., temperature) were the most beneficial for storage stability (Table 1). The starting titres were 2.76 × 10^9^ ± 4.28 log_10_ CFU mL^−1^ and 3.67 × 10^9^ ± 2.93 log_10_ CFU mL^−1^ in Middlebrook and Roisin’s respectively. The analyses showed that +4 °C was the optimal storage temperature regardless of its pairing with other conditions; mitigating the reduction in log_10_ CFU mL^−1^ loss, in all conditions, to 0–0.2 log_10_ CFU mL^−1^ (Figure 3). When stored at −20 °C or −80 °C, there was a significant decrease of 0.9–1.3 log_10_ CFU mL^−1^ compared to storage at +4 °C, though when paired with 1.5% MSG supplemented with either DMSO or glycerol, the significant difference was lost and the reduction in viable counts was 0.2–0.6 log_10_ CFU mL^−1^. The DMSO-supplemented 1.5% MSG solution gave the most effective level of cryoprotection at −20 °C and −80 °C, mitigating against any significant decrease in the log_10_ CFU mL^−1^ between the three storage temperatures (*p* value = 1.000 for all comparisons). The cryoprotectant, 1.5% MSG supplemented with glycerol, also demonstrated a protective effect against the freeze-thaw process. However, it was not as effective as DMSO as it did not pass the significance threshold when comparing it to MSG alone. Glycerol supplementation appeared to have had a marginally reduced protective effect when compared to DMSO, although, this isn’t significant, and with glycerol there was no significant difference in the total viable counts between −20 °C and −80 °C *(p* value = 1.000, 0.8222 & 0.7617). The medium used did not appear to impact CFU mL^−1^ retained post-storage. Although Middlebrook appeared to have been marginally more detrimental with a longer culture duration, there was no significant difference between media used (*p* value = 0.1291). Sampling after 4 days demonstrated optimal retention of viable bacteria, with CFU mL^−1^ only dropping by 0.5–1.0 log_10_ CFU mL^−1^ as culture duration increased, regardless of paired conditions.

## 4. Discussion

In this study, we demonstrate the ability to cultivate *M. bovis* BCG in controlled batch fermenters under reproducible conditions to a high titre while ensuring that culture viability is maintained. We have shown that a complex medium such as Middlebrook or a minimal medium such as Roisin’s provide comparable growth. In parallel, fermenter-growth of BCG in Middlebrook has demonstrated protection against *M. bovis* in badgers [17]. This and other studies have highlighted the benefits of fermentation for generation of the BCG vaccine to high titres and should be considered as an attractive alternative to pellicle growth for animal and/or human BCG vaccine production [13]. Fermentation would be a quicker, more controlled, and reproducible alternative to pellicle-growth for BCG production, with the added benefits of future standardisation. Dietrich et al., [13] relied on pellicle growth in Sauton’s for preparation of their inoculum and used the same medium in their fermentations [13]. We used a simpler, quicker, consistent, shaking flask method for growth of the inoculum, but curiously *M. bovis* BCG would not grow in Sauton’s using this approach; or in Sauton’s in a fermenter. It is possible that for *M. bovis* BCG to grow in Sauton’s it first needs to be passaged and adapt in a clumpy biofilm-like state in static cultures prior to growth in stirred or shaking cultures. This raises concerns about the impact of multiple culture steps on the genetic stability of the strain. *M. bovis BCG* also clumps during growth in fermenters. The inclusion of detergent in culture is a necessity for generating single cells or smaller clumps of bacilli and generates a more homogenous culture [13,18]. However, the presence of detergent in the growth medium can affect the capsule composition of BCG and this is associated with differences in the outcome of vaccination efficacy, implying that these are important factors in immunological studies [24]. Tween 80 was present in our culture media at a low concentration, and although we haven’t compared protection with or without Tween 80, protection was afforded by fermenter-grown BCG in Middlebrook [17]. It has also been shown that preparation of homogeneous suspensions of BCG from cultures grown in Sauton’s by the chemical action of Tween 80, gave higher concentrations of viable bacteria than the ball-milling method traditionally used [25]. Of the two media selected for fermentation in our study, there were no significant differences detected between them; both supporting the propagation of BCG to a comparable level. Middlebrook is a complex, undefined, medium and is readily available worldwide, whilst Roisin’s is a minimal medium that is defined and notably cheaper to produce than Middlebrook (Middlebrook costs ~£83/L to produce whilst Roisin’s costs ~£2/L; at the time of writing). Roisin’s was first described by Beste et al., [26] for the growth of *M. bovis* BCG in chemostat fermenters; it can be manipulated, and quantitative substitutions made for individual medium components, which creates an opportunity for further optimisation of the medium for BCG production by fermentation. Comparative studies would need to be carried out between *M. bovis* BCG cultivated via traditional methods and fermentation in Roisin’s, including the impact of larger scale fermentation on the cultivation of BCG and protection/virulence studies in animal models.

Fermentation provides a more uniform, reproducible approach than pellicle growth and leads to a less clumpy, more homogenous population. This lends itself to quantification by alternative approaches, such as flow cytometry, which can provide information about the total number of bacilli in the BCG vaccine and the metabolic status of the bacterial population. Current BCG production methods use pellicle growth, which leads to a heterogenous population of bacteria, the formation of bacterial aggregates, and a high proportion of dead bacilli. These factors are likely to result in inconsistent batches of BCG vaccine, which is a challenge for manufacturers in their quality control processes. We applied our previously published, rapid, flow cytometry method that employs the use of fluorescent dyes to determine percentages of live or dead bacteria in culture. Cell samples were dual-stained using calcein violet with an acetoxy-methyl ester group (CV-AM; measures metabolic activity) and sytox green (SG; measures cell wall permeability, a proxy for cell death) followed by flow cytometry. Flow cytometry has become a useful technology in understanding the physiology of *M. tuberculosis* during TB disease, particularly population heterogeneity [27]. This approach has the advantage of capturing information about the metabolic status of the whole population, whereas colony counting is an indirect measure of population death, quantifies only viable bacteria that are culturable on agar, and does not generate information about organisms that are not immediately culturable. Flow cytometry is also rapid and can be performed within the life of the culture, instead of having to wait three weeks of plate incubation for colony counts. Previously, Dietrich et al. [13] determined the viability of fermenter-grown *M. bovis* BCG by fluorescence labelling with Fluorassure reagent, which has a similar mechanism of action to CV and consists of fluorogenic esters which are hydrolysed by esterases present only in live bacteria, leading to the accumulation of fluorescent product in viable bacterial cells [28,29]. Fluorescent signals from viable bacteria were measured with a Chemscan. Our method has the added advantages of directly measuring the proportion of dead bacteria in the whole population. We have shown here, that the culture method used does not lead to an increase in the proportion of dead bacteria over time, during the 12 days of incubation. Total cell counts could be used as an early indication of total viable counts and provide a more accurate enumeration of bacilli in the BCG vaccine as it counts bacilli that are present in the culture but cannot be cultured on agar. Flow cytometry has been used previously to enumerate bacteria, with an accurate reflection of viable bacterial load [30,31,32]. This is the first time that flow cytometry has been used for population-wide analyses of the metabolic status of fermented *M. bovis* BCG. Combined with the other methods described here, flow cytometry has allowed for a direct comparison between total cells, culturable bacteria, dead bacteria, and metabolically active bacteria, particularly as the same technology, flow cytometry, is used for all parameters apart from colony counts. Further studies will focus on generating large quantities of data so that the relationships between these factors can be mathematically modelled to provide predictive formulae for early predictions of batch quality and standardisation of the BCG vaccine.

A further important consideration for optimal BCG production is the storage of BCG vaccine post-culture to retain high numbers of viable bacilli [9]. Although lyophilisation is the standard for BCG vaccine preparation, lyophilisation of BCG can result in as much as a 50% reduction in live bacilli [33]. Alternative methods of storage may be more compatible with wildlife bait vaccination programs and important for many other applications, such as the stable maintenance of aerosol challenge stocks of pathogenic bacteria for vaccine efficacy studies. We evaluated the combined impact of three cryoprotectant solutions at different temperatures, compared with MSG, which has been shown to have the highest effectiveness on the stability of the vaccine [34,35]. Vaccine containing 1% MSG was characterised by best thermostability, homogeneity, and the high survival of bacilli during freeze-drying [9,36,37,38,39]. Wong et al., in 2007 [40], demonstrated that BCG suspended in 1.5% MSG could be dried without freezing, through spray drying, and maintained stably at room-temperature for several months. Supplements to MSG, 10% glycerol and 5% DMSO, were assessed here. Glycerol is a known cryoprotectant and complete survival of BCG vaccine was obtained previously by Gheorghiu et al., in 1996, whereby the BCG bacilli were frozen in a glycerol solution and stored at −70 °C for several years [41]. Both glycerol and DMSO previously showed protective benefits at low temperatures in a study by Grover et al., in 1967, and the results of our study reflect these findings [42]. Survival at +4 °C is significantly higher, here, but can only be sustained for up to three months, which could be impractical. Both supplements used, reduced the impact of a low storage temperature, with DMSO having a more significant, positive effect, on survival.

## 5. Concluding Remarks

We have shown that *M. bovis* BCG can grow to a high titre in Roisin’s, which is a cheap, defined, medium that has utility in the fermentation of BCG vaccine. The use of 1.5% MSG containing 5% DMSO appears to be a promising cryoprotectant to improve *M. bovis* BCG survival at low storage temperatures after production. Fermentation of BCG combined with real-time measurements of culture titre, and metabolic state, by using flow cytometry, has the potential to improve the standardisation and quality control of commercial BCG production.

## Figures and Tables

**Table 1:** The *p*-values of pair-wise comparisons for individual conditions (e.g., −20 °C, −80 °C, or +4 °C) within each parameter (e.g., temperature) that were determined to be significant by factorial ANOVA. The pair-wise comparisons were generated by a post-hoc Tukey’s HSD Test on cube-root transformed CFU mL^−1^; the condition that is most favorable for retention of total viable counts is the first condition of each comparison.

Pair-Wise Comparison	*p*-Value
Temperature	
+4 °C vs. −80 °C	0.0002 ***
+4 °C vs. −20 °C	0.0022 **
−20 °C vs. −80 °C	0.7163
Cryoprotectant	
5% DMSO vs. 1.5% MSG	0.0003 ***
10% Glycerol vs. 1.5% MSG	0.0698
5% DMSO vs. 10% Glycerol	0.1535

*** *p* = 0.001; ** *p* = 0.01.

## Supplementary Materials

The following are available online at https://www.mdpi.com/1999-4923/12/9/900/s1, Figure S1: The total viable counts of *M. bovis* BCG flask grown in 50 mL of CMM MOD2, Proskaur-Beck and Dorset-Henly over 14 days. Figure S2: The total viable counts of *M. bovis* BCG (fermenter grown in Middlebrook 7H9) over time stored at +4 °C, −20 °C, and −80 °C. Table S1: The *p*-values of factors medium, storage temperature, cryoprotectant and culture duration, with interactions tested.
