# Supplementary material for: Optimisation of Mycobacterium bovis BCG Fermentation and Storage Survival
Source: Pharmaceutics. 2020 Sep 22;12(9):900. doi: 10.3390/pharmaceutics12090900 (PMC7558299; doi:10.3390/pharmaceutics12090900)
Supplement: Supplementary file 1 [file pharmaceutics-12-00900-s001.pdf]

Supplementary Materials: Optimisation of *Mycobacterium bovis* BCG Fermentation and Storage Survival

Jordan Pascoe, Charlotte L. Hendon-Dunn, Colin P.D. Birch, Gareth A. Williams, Mark A. Chambers and Joanna Bacon

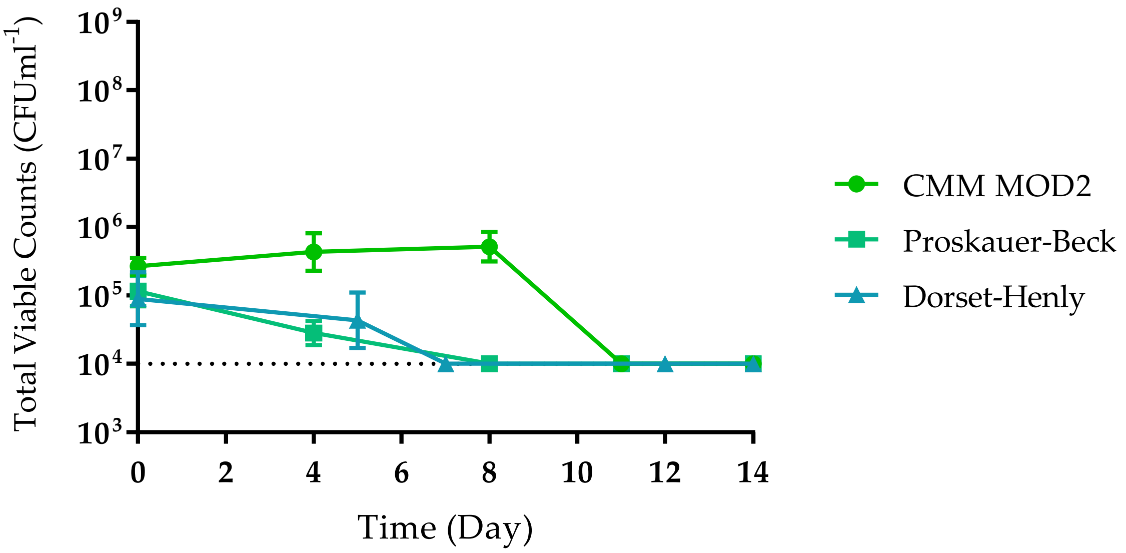

Figure S1. The total viable counts of *M.bovis* BCG flask grown in 50 mL of either CMM MOD2, Proskaur-Beck or Dorset-Henly, over 14 days. Cultured in 250 mL vented Erlenmeyer flasks shaking at 200RPM at 37°C. 100µl samples were taken, a 10-fold dilution series performed and spotted onto 7H10 + 10% OADC agar using the Miles and Misra method. Plates were incubated for three weeks at 37°C and then colonies enumerated. Dotted line represents the limit of detection. Data represents the mean average of three biological repeats ± standard error.

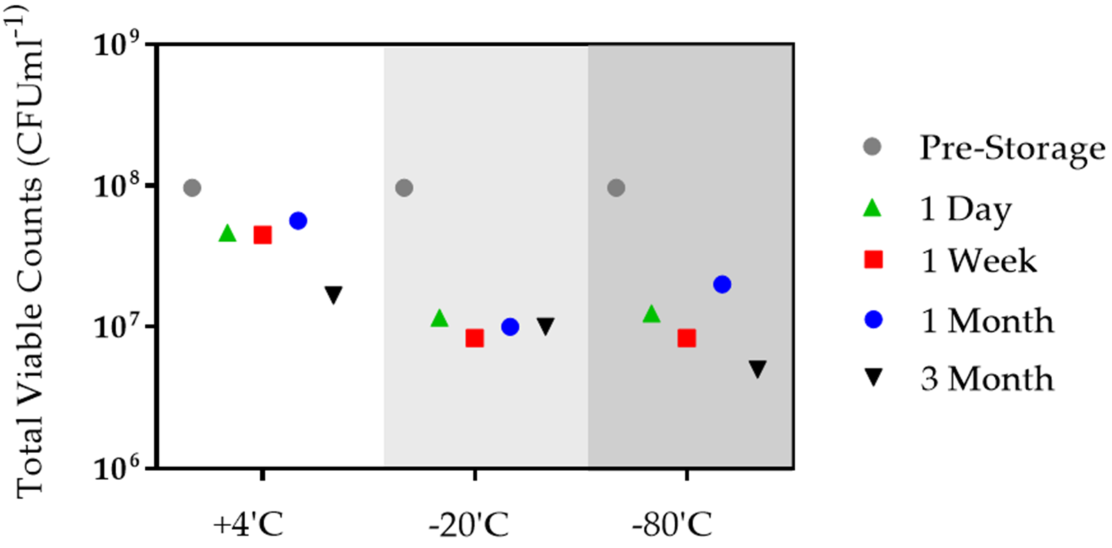

Figure S2. The total viable counts of *M.bovis* BCG (fermenter-grown in Middlebrook 7H9) over time stored at +4°C, −20°C, and −80°C. A 20ml sample was taken from the fermenter grown M.bovis BCG after 12 days of culture. The sample was spun at 3750RPM, supernatant removed and resuspended in 20ml of 1.5% MSG (w/v in water). Triplicate samples of 100µl were then taken to assess pre-storage total viable counts via a 10-fold dilution series, performing a modified Miles and Misra method. 100µl samples were stored at at +4°C, −20°C, and -80°C, and then an individual sample removed after one day, one week, one month and three months for enumeration via a modified Miles and Misra method. Data represents a single biological replicate.

Table S1. Recipe of Middlebrook 7H9 [1].

Component	Amount g/L
L-glutamic acid	0.5
Glycerol	5 (mL)
Tween 80	2 (mL)
Disodium Phosphate	2.5
Monopotassium Phosphate	1
Magnesium Sulphate	0.05
Calcium Chloride	0.0005
Ammonium Sulphate	0.5
Sodium Citrate	0.1
Oleic Albumin Dextrose Catalase (OADC)	100(mL)
Ferric ammonium Citrate	0.04
Zinc sulphate	0.001
Copper Sulfate	0.001
Pyridoxine	0.001
Biotin	0.0005

[1] Medium prepared from Middlebrook 7H9 broth base bottle (BD Difco™).

Table S2. Recipe of Sauton's minimal medium.

Component	Amount g/L
L-Asparagine	4
Glycerol	60 (mL)
Tween 80	2 (mL)
Monopotassium Phosphate	0.5
Magnesium Sulphate	0.5
Citric Acid	2
Ferric Ammonium Citrate	0.05

Table S3. Recipe of Roisin's minimal medium.

Component	Amount ml/L
Glycerol	5
Tween 80	2
×10 Basic Salts Solution	100
1 M Calcium Chloride	0.5
1 M Magnesium Chloride	0.5
×1000 Trace Element Solution	1

Table S4. Recipes of the ×10 Basic Salt Solution and ×1000 Trace Element Solution used in Roisin's minimal medium.

×10 Basic Salt Solution	Amount g/1L
Ammonium Chloride	59
Disodium Phosphate	25
Potassium Phosphate monobasic	10
Potassium Sulphate	20
**×1000 Trace Elements Solution**	**Amount mg/1L**
Zinc Chloride	80
Iron(III) Chloride	400
Copper(II) Chloride	20
Manganese(II) Chloride	20
Sodium Tetraborate Decahydrate	20
Ammonium Molybdate Tetrahydrate	20

Table S5. Recipe of CMM MOD2 medium.

Component	Amount g/L
L-asparagine	2
L-serine	0.1
L-alanine	0.1
L-arginine	0.1
L-aspartic acid	0.1
L-glycine	0.1
L-isoleucine	0.1
L-leucine	0.1
L-glutamic acid	0.1
Glycerol	0.75
Tween 80	2 (ml)
ACES Buffer	10
Monopotassium Phosphate	0.22
Sodium Bicarbonate	0.042
Magnesium Sulphate	0.214
Calcium Chloride	0.00055
Pyruvic Acid Sodium Salt	1
Zinc Sulphate	0.0288
Iron Sulphate	0.01
Cobalt Chloride	0.00048
Copper Sulfate	0.000025
Manganese Chloride	0.00002
Biotin	0.0001

Table S6. Recipe of Proskauer-Beck medium.

Component	Amount g/L
Glycerol	20.0 (mL)
Tween 80	2 (mL)
L-Asparagine	5.0
Magnesium Citrate	2.0
Monopotassium Phosphate	5.0
Magnesium Sulphate	0.6

Table S7. Recipe of Dorset-Henly medium.

Component	Amount g/L
Glycerol	8 (mL)
Glucose	10
Tween 80	2 (mL)
L-Asparagine	14
Sodium Citrate	0.74
Monopotassium Phosphate	1.5
Magnesium Sulphate	1.5
×100 Trace Element Solution	1 (mL)
Ferric ammonium citrate	0.681

Table S8. Recipe of the x100 Trace Element solution used in Dorset-Henly medium.

×100 Trace Element Solution	Amount g/0.4L
Zinc Sulphate	8
Manganese Chloride	0.8
Cobalt Chloride	0.4

Table S9. Factorial ANOVA was performed on the changes in the total viable counts (CFU/mL) between pre- and post-storage for the combined parameters to assess whether individual parameters or interactions between parameters exhibited a statistically significant impact on *M. bovis* BCG stability. '***' $p = 0.001$, '*' $p = 0.05$.

Factors	*p*-value
Temperature	0.0001 ***
Cryoprotectant	0.0004 ***
Temperature:Cryoprotectant	0.0442 *
Day	0.0951
Media	0.1165
Media:Temperature:Day	0.1673
Media:Day	0.2041
Media:Temperature	0.4096
Media: Cryoprotectant	0.6212
Temperature:Day	0.9525
Media:Temperature: Cryoprotectant	0.9656
Media:Cryoprotectant:Day	0.9943
Cryoprotectant:Day	0.9968
Media:Temperature: Cryoprotectant:Day	0.9973
Temperature: Cryoprotectant:Day	0.9993
